# Serotonin's Role in the Pancreas Revealed at Last

**DOI:** 10.1371/journal.pbio.1000227

**Published:** 2009-10-27

**Authors:** Richard Robinson

**Affiliations:** Freelance Science Writer, Sherborn, Massachusetts, United States of America

**Figure pbio-1000227-g001:**
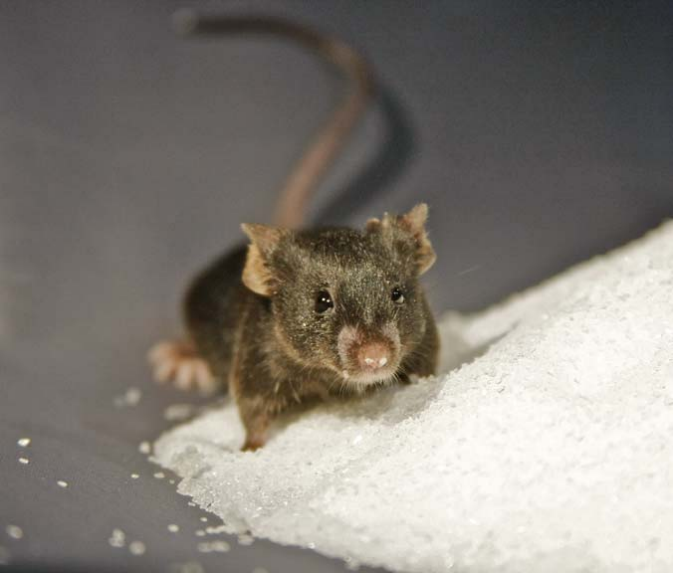
Sugar and mice that were unable to make serotonin helped identify a link between diabetes mellitus and the “feel-good” hormone serotonin.

[Fig pbio-1000227-g001]Serotonin is best known as a neurotransmitter in the brain. Released by one neuron and picked up by another, it regulates mood and aggression. Many antidepressants, and the party drug Ecstasy, increase serotonin stimulation. But it is found outside the central nervous system as well, including in the pancreas, specifically in the beta cells that release insulin to regulate blood glucose levels. Serotonin was discovered in beta cells over 30 years ago, and researchers have been trying to figure out what it's doing there ever since. In a new study, Nils Paumann, Diego Walther, and colleagues show that serotonin plays a key role in controlling insulin secretion and that its absence leads to diabetes. Even more surprisingly, it does so through a completely different mechanism than that used in the brain—it acts within, not between, cells and, rather than forming brief and weak liaisons with a receptor, makes long-lasting covalent bonds with an enzyme.

When released from the pancreas, insulin helps other cells in the body take up sugar from the bloodstream, feeding the cells and bringing blood sugar levels down again. Diabetes, in its several forms, arises when the system goes awry. Before its release, insulin is stored in beta cells in secretory granules. These granules also store serotonin, which is released along with insulin. (In fact, when cell biologists want to track insulin release, they often measure serotonin instead because it's easier to monitor.)

Prior studies have offered some clues to serotonin's activities in other tissues in the body's periphery. In platelet-forming cells called thrombocytes, it links up to a group of signaling enzymes called GTPases, triggering the release of vesicle contents by exocytosis. Unlike the fleeting connections it makes to receptors within the brain, serotonin covalently bonds to the GTPase, a reaction called “serotonylation,” which is catalyzed by a transglutaminase enzyme. Intriguingly, GTPases also help regulate insulin secretion in the pancreas, and lack of transglutaminase leads to glucose intolerance, both suggesting that the same system may be at play in the pancreas.

To test this directly, the authors examined mice lacking tryptophan hydroxylase, a key enzyme in serotonin synthesis, rendering the mice unable to make serotonin outside the central nervous system. The mice exhibited classic signs of diabetes—elevated blood glucose and decreased insulin secretion. They were also resistant to the effects of pargyline, a chemical that in normal mice causes insulin release, suggesting that the absence of serotonin blocked this effect. Infusion of serotonin normalized secretion in mice unable to make their own.

The authors found that serotonin in pancreatic cells bound directly to GTPase enzymes, and by blocking transglutaminase, blocked this reaction, reducing insulin secretion. The authors identified two specific GTPases that are known to play a role in insulin secretion as targets for serotonylation. But, based on the large amount of protein-bound serotonin they saw, they suggest serotonin likely links up with multiple other proteins as well, suggesting a host of other interactions yet to be discovered.

The model that emerges from this study suggests that by binding to GTPases, serotonin promotes insulin release in response to elevated glucose. In addition to solving the puzzle of what a brain neurotransmitter is doing in the pancreas, the study has some important clinical implications. The condition that developed in the mice lacking tryptophan hydroxylase most resembled the human form of disease known as “maturity onset diabetes of the young,” raising the intriguing prospect that this disease may be linked to mutation of the tryptophan hydroxylase gene, a possibility that will require further study. Whether or not this is so, the serotonin pathway provides an avenue for intervention in multiple forms of diabetes, since it directly influences the amount of insulin secreted. Given the medical importance of diabetes—it affects almost 200 million people worldwide, and requires chronic treatment—it is a good bet that serotonin modulation will soon be a target for drug development.

**Paulmann N, Grohmann M, Voigt J-P, Bert B, Vowinckel J, et al. (2009) Intracellular Serotonin Modulates Insulin Secretion from Pancreatic β-Cells by Protein Serotonylation. doi:10.1371/journal.pbio.1000229**

